# Recurrent bilateral brachial plexus neuritis following rapid semaglutide-induced weight loss: a case report

**DOI:** 10.1186/s12883-026-04664-4

**Published:** 2026-01-26

**Authors:** Lukács S. Lesinszki, Radmanesh Khalili, Haiyang Jiang, Shivangi Jha, Peter G. Bernad

**Affiliations:** 1 Department of Physiology, Semmelweis University School of Medicine, Tűzoltó utca 37-47, Budapest, 1094 Hungary; 2 Department of Neurology, Karl Landsteiner University of Health Sciences, Vienna, Austria; 3 Tongji Medical College of Huazhong, University of Science and Technology, Wuhan, China; 4 Albert Szent-Györgyi Medical School, University of Szeged, Szeged, Hungary; 5 Department of Neurology and Rehabilitation Medicine, George Washington University, Washington D.C, United States of America

**Keywords:** Brachial plexus neuritis, GLP-1 agonists, Semaglutide, Case report

## Abstract

**Background:**

Brachial plexus neuritis, or Parsonage–Turner syndrome, is an uncommon neuropathy marked by acute pain followed by weakness, often triggered by infection, vaccination, trauma, or metabolic stress but the exact cause of this disease remained unknown. Semaglutide is widely used for the treatment of type 2 diabetes and obesity. With its increasing use, there have been reports suggesting a possible association with neuropathies. Currently, there is no information suggesting a possible association between the use of semaglutide and brachial plexus neuritis.

**Case presentation:**

A 39-year-old man with a prior episode of unilateral brachial neuritis developed recurrent bilateral upper limb weakness after an eight-month, 70-pound semaglutide-associated weight loss. He presented with bilateral radial sensory loss, finger extensor paralysis, right triceps weakness and decreased grip strength on the left side. Corticosteroids provided pain relief, but motor and sensory deficits persisted despite physiotherapy.

**Conclusion:**

This case highlights a temporal association between rapid pharmacologic weight loss and recurrent idiopathic brachial plexus neuritis, emphasizing the importance of gradual, nutritionally supported weight reduction and vigilance for neuromuscular complications during GLP-1 receptor agonist therapy.

## Background

Brachial plexus neuritis (BPN), also known as idiopathic brachial neuritis, neuralgic amyotrophy, or Parsonage-Turner syndrome, is an uncommon peripheral nerve disorder of uncertain etiology that typically involves asymmetric inflammation of the brachial plexus [1, 2]. The condition usually presents in young to middle-aged male adults with the sudden onset of severe shoulder or upper arm pain lasting several days to weeks, followed by painless weakness and variable sensory disturbances in the affected limb [3]. Recovery is slow and often incomplete, with a significant proportion of patients experiencing long-term deficits. Although the clinical diagnosis is usually straightforward in typical cases, atypical presentations or lack of physician familiarity frequently lead to misdiagnosis. Electromyography and nerve conduction studies, magnetic resonance imaging (MRI) of the shoulder or brachial plexus, and cerebrospinal fluid analysis are not required for diagnosis, but can provide supportive evidence and help rule out alternative conditions [2, 4].

The etiology of BPN remains incompletely understood, but the most widely accepted hypothesis suggests an immune-mediated attack on the brachial plexus or its peripheral branches, often triggered by preceding events such as infections, vaccinations, trauma, or metabolic stress [5, 6]. Pathological evidence is limited, though a single report of brachial plexus biopsy demonstrated mononuclear inflammatory infiltrates, supporting an inflammatory mechanism [7]. While the majority of cases are sporadic, a hereditary form—hereditary neuralgic amyotrophy (HNA)—has also been described. HNA can follow an autosomal dominant inheritance pattern linked to chromosome 17q24 and the mutation of septin-9 (SEPT9) and is characterized by recurrent attacks precipitated by the same triggers as the idiopathic form, though it may exhibit additional distinct clinical features [5, 8].

Semaglutide, a glucagon-like peptide-1 (GLP-1) receptor agonist, has been shown in large randomized clinical trials (SUSTAIN 1–5) to induce clinically significant weight loss, with mean reductions ranging from 2.3 to 6.3 kg compared with placebo or active comparators over 30–56 weeks, leading to unprecedented uptake worldwide [9]. While its cardiometabolic benefits are substantial, rapid pharmacologic weight reduction may mimic the neuro-metabolic stress augmentation as seen in bariatric patients [10, 11]. Emerging evidence has raised concern about neuropathic complications associated with semaglutide and other GLP-1 receptor agonists. Several studies describe an association with nonarteritic anterior ischemic optic neuropathy (NAION), now recognized as a very rare adverse event [12, 13]. There is also evidence that diabetic lumbosacral radiculoplexus neuropathy and common fibular neuropathy have been reported in association with GLP-1 receptor agonist use [14]. These observations suggest that metabolic stress and rapid weight reduction caused by GLP-1 receptor agonists can lead to neurological complications, emphasizing the importance of further surveillance.

### Case presentation

**Fig. 1:**
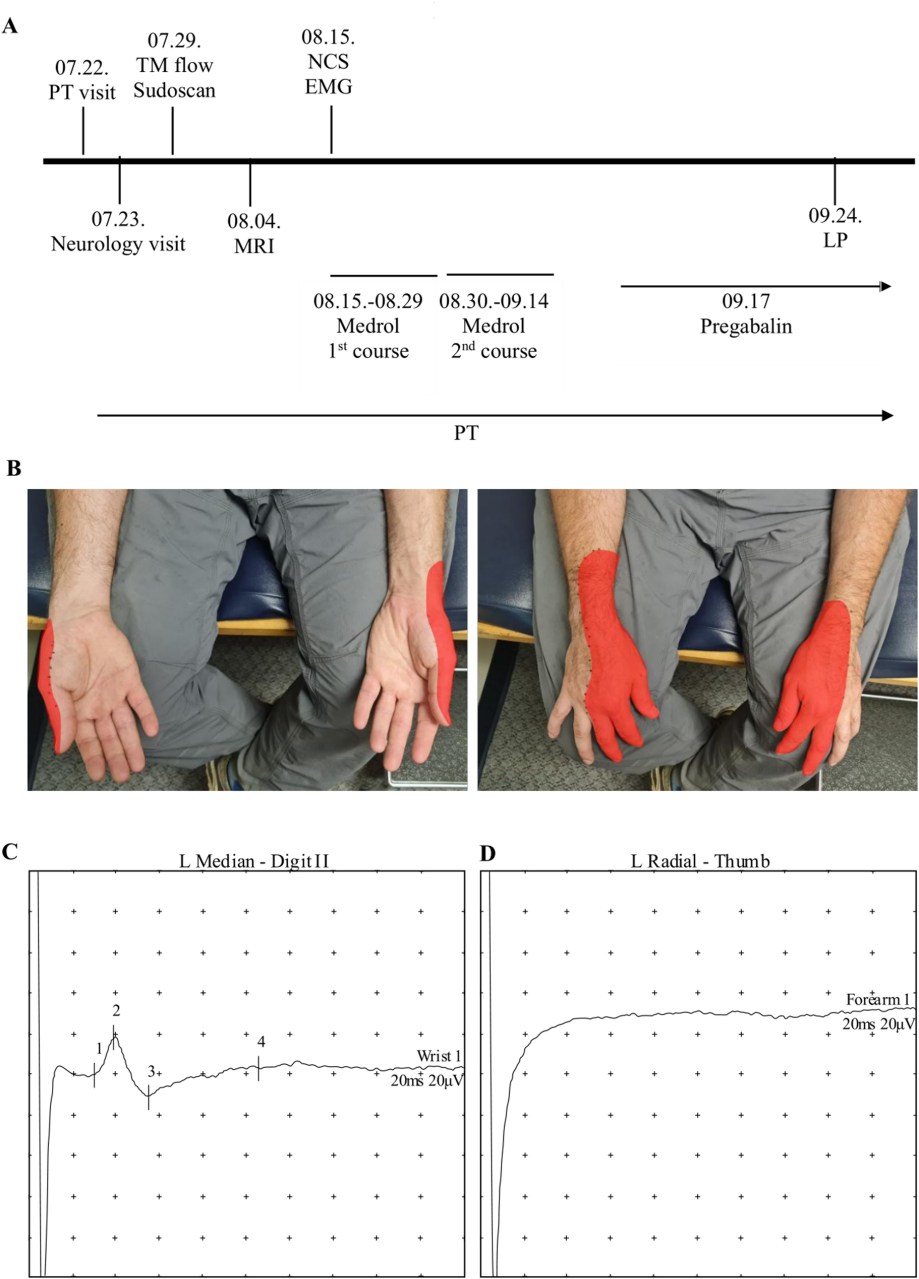
**A** Timeline of the diagnostic evaluations, and therapeutic interventions in 2025. **B-D**, Examination of sensory nerve function. **B** The distribution of the sensory deficit in the patient is shown in red. Sensory examination was performed using a Wartenberg wheel and cotton wool. **C-D** Representative sensory nerve action potentials (SNAP) in the left median (**C**) and radial nerve (**D**). The median nerve was stimulated at wrist and recorded at digit II, while the radial nerve was stimulated at the forearm and recorded at the thumb

A 39-year-old right-handed male electrician presented in 23rd of July 2025 with sudden-onset bilateral upper extremity weakness and pain (Fig. 1A). His bilateral arm weakness started around 27th of June, but his arm pain started around one month before. In December 2024, he started taking semaglutide for weight management, which was the only medication he was on. The dose of semaglutide gradually increased to 2.4 mg weekly. Over the following eight months, he lost approximately 70 pounds, decreasing from 255 lbs (116 kg) to 185 lbs (84 kg). He denied intercurrent infections, recent vaccinations, systemic illnesses, or trauma during this period. However, in May 2025, he reported diffuse body pain following excessive consumption of daily supplements, including vitamin B100 complex, L-arginine, zinc, and energy drinks containing 6 µg vitamin B12 and 30 mg niacin per serving; the symptoms resolved promptly after discontinuation. Family and social history were unremarkable. In his past medical history, eczema and occasional episodes of elevated blood pressure were noted, for which he was not taking any medication in 2025. He had no history of major surgeries or other chronic illnesses. Past infectious screening, including an STD panel during an infertility evaluation was negative. His occupational history included frequent lifting of heavy loads and overhead work, but he denied recent physical injury or electrical accidents.

His symptoms began with sudden, severe bilateral hand pain, described as deep, burning, and radiating proximally, graded 7/10. He started taking ibuprofen and his primary care provider (PCP) initially commenced him on gabapentin, but it provided no relief. Within 1 month, he developed progressive numbness and weakness. The patient initially attributed the symptoms to carpal tunnel syndrome due to distal sensory involvement.

In 2019, he developed severe left shoulder pain followed by weakness. Physical examination revealed winging of the left scapula, weakness in external rotation of the left upper arm, and weakness of the left triceps. The remainder of the physical examination, including assessment of sensory function, cerebellar function, cranial nerves and reflexes was unremarkable. MRI demonstrated an intact brachial plexus and cervical spine, with feathery edema consistent with denervation edema in the infraspinatus, teres minor, and posterior deltoid muscles. The remainder of the musculoskeletal structures of the shoulder, including the tendons, ligaments, bursa, and glenoid were intact. Electromyography (EMG) and electroneurography (ENG) showed normal sensory and motor nerve conduction in both upper and lower extremities; however, nerves innervating the shoulder girdle muscles were not assessed. Overall, the clinical presentation was suggestive of BPN. The episode resolved with supportive management, and physical therapy (PT) leaving no lasting neurological deficits. The strength of his arm completely returned after 1.5 years after this episode.

On recent examination, he had a complete inability to extend the fingers of either hand, significant weakness of the right triceps with intact strength in the left triceps, mainly preserved shoulder and deltoid strength bilaterally, winging of the right scapula, and decreased grip strength on the left side. Sensory assessment revealed decreased sensation in the radial nerve distribution bilaterally on his distal upper extremities (Fig. 1B). Reflexes showed a normal left biceps reflex, while the right biceps reflex was not elicitable due to tenderness at the cubital fossa. Lower extremity reflexes were symmetric and intact. Special tests for upper motor neuron signs, including Hoffmann’s and Tromner’s, were negative, as was Froment’s sign. Muscle atrophy was not visible in either extremity. Cerebellar testing, gait assessment, and cranial nerve examination were all normal.

MRI of the cervical spine and brachial plexus revealed an intact brachial plexus with no T2 hyperintensity. C3-C4 showed minimal disc desiccation, C5-C6 a small central disc protrusion (old), and C6-C7 a left paracentral disc protrusion with minimal narrowing of the left lateral recess. Spinal cord was intact with no sign of demyelination. TM flow and Sudoscan tests were normal, making small fiber neuropathy unlikely. Electromyography and nerve conduction studies showed normal sensory and motor conduction velocities in the median and ulnar nerves, but radial nerve sensory and motor signals could not be obtained on either side (Fig. 1C, D). Needle EMG revealed some giant potentials in the C7–T1 muscle distribution of the left upper extremity, with otherwise normal findings in upper and lower extremities. Lumbar puncture (LP) revealed an opening pressure of 19 cm H₂O and a closing pressure of 15 cm H₂O. CSF was clear, with glucose 51 mg/dL, protein 43 mg/dL, and no leukocytes or bacterial growth. Acute brachial diplegia could be a rare manifestation of Lyme disease [5, 15]; however, to exclude this possibility, CSF PCR for *Borrelia* species was performed and was negative. The level of myelin basic protein in CSF (MBP) was also normal, making a demyelinating pathology such as multiple sclerosis (MS) unlikely.

Based on the acute pain, subsequent motor weakness, focal neurological findings, and prior history of brachial neuritis, the presentation was consistent with recurrent BPN. The temporal relationship with rapid, pharmacologically induced weight loss suggested a possible metabolic or immune-mediated trigger. As part of the therapy, semaglutide was discontinued at first visit. Methylprednisolone therapy was initiated after diagnosis (56 mg oral methylprednisolone with a decreasing dose over 14 days), leading to partial improvement in pain (reduced to 3/10), but motor and sensory deficits persisted after two weeks. Another course of methylprednisolone therapy was started. At the end of the last steroid course, 150 mg/day pregabalin therapy was initiated. Physical therapy partially restored hand function, though bilateral weakness remained disabling.

## Discussion

This case highlights a recurrent and atypical presentation of BPN temporally associated with semaglutide-induced rapid weight loss. The recurrence and atypical, bilateral presentation in this patient, coinciding with significant pharmacologic weight loss, suggest that rapid metabolic shifts may represent a novel precipitant of BPN.

The mechanisms by which weight reduction might increase vulnerability to neuropathy are likely multifactorial. Nutritional deficiencies such as thiamine, vitamin B12, or copper are well-recognized causes of neuropathy and have been described in patients after bariatric surgery [11]. However, the patient reported consuming supplements (daily vitamin B100 complex, zinc, L-arginine); therefore, dietary restriction is less likely to have contributed. In addition, metabolic stress may trigger immune dysregulation, with cytokine-mediated inflammatory responses directed against peripheral nerves. The loss of perineural fat with rapid weight loss may also reduce mechanical protection, increasing susceptibility to traction, compression or twisting in physically active individuals. These factors may converge to precipitate brachial plexus inflammation in predisposed patients.

Bilateral radial nerve involvement is rare in brachial plexus neuritis; however, a case report described that twisting of the radial nerves was implicated in the development of this condition [16]. In our case, MRI did not reveal any signs of twisting, such as the hourglass sign, possibly because the twisting had resolved by the time of imaging or because the affected segment of the radial nerve was too distal to be captured.

Differential diagnosis included cervical spondylotic amyotrophy, but it this case sensory involvement is not present [17]. The presence of only lower motor neuron signs and peripheral sensory nerve changes makes ALS unlikely. MRI excluded compression or traumatic injury of the brachial plexus. The LP and MRI findings, along with the physical examination, provided no evidence of multiple sclerosis or other demyelinating disorders.

Bilateral finger extension weakness, sensory loss in the radial nerve distribution, and right-sided triceps weakness, together with the ENG findings, support bilateral radial nerve injury. Decreased grip strength on the left side and denervation potentials in the flexor digitorum superficialis muscle may indicate partial median nerve involvement; however, the affected branches were not sampled by ENG. Winging of the right scapula might represent long thoracic nerve injury. A comprehensive ENG or EMG examination was not performed to determine the exact localization of the injured branches; however, the simultaneous and asymmetric involvement of multiple nerves, based on clinical presentation, is more consistent with brachial plexus neuritis than with other peripheral neuropathies.

Although semaglutide has demonstrated substantial cardiometabolic benefits, recent literature has drawn attention to neurological adverse effects. Case reports and pharmacovigilance analyses have highlighted NAION as a rare but serious complication of GLP-1 receptor agonist therapy [12, 13]. Pharmacovigilance perspectives emphasize that, while current evidence is conflicting, transparent and reproducible safety monitoring is essential to clarify emerging risks [18].

From a diagnostic perspective, BPN remains a clinical diagnosis characterized by the reproducible sequence of acute pain followed by weakness and sensory loss. Investigations such as MRI and EMG serve primarily to exclude alternative etiologies, including cervical radiculopathy, compressive lesions, or generalized neuropathy. The clinical presentation in this patient, bilateral but asymmetric upper extremity involvement, recurrence, is most consistent with Parsonage–Turner syndrome.

Possible confounders that could have precipitated the recurrence of BPN, besides rapid weight loss, include excessive consumption of pyridoxine and caffeine in May, which might have caused metabolic alterations. The patient also continued working as an electrician, performing overhead work, which could act as an additional confounder.

Taken together, this case may expand the spectrum of potential adverse neurological effects of semaglutide by raising the possibility that rapid pharmacologic weight loss may contribute to the development of BPN. Clinicians should remain vigilant for neuromuscular complications in patients prescribed GLP-1 receptor agonists and encourage gradual, nutritionally supported weight reduction strategies. Early recognition and supportive management remain key to optimizing outcomes in this rare but potentially disabling condition.

## Conclusion

This case illustrates a recurrence of BPN coinciding with rapid, semaglutide-induced weight loss. The sequence of acute pain, subsequent weakness, and focal neurological deficits was consistent with Parsonage–Turner syndrome, while the close temporal link with weight reduction raise the possibility of a pharmacologically mediated precipitant. Further translational and clinical studies are needed to elucidate the molecular mechanisms by which semaglutide-associated weight loss might contribute to the development of BPN. Although causality cannot currently be proven, this is, to our knowledge, the first case report describing this possible adverse effect of semaglutide. This observation highlights the need for clinician awareness of potential neuromuscular risks associated with GLP-1 receptor agonist therapy.

## Data Availability

The data supporting the conclusions of the study are available from the corresponding author upon reasonable request.

## Abbreviations

ALS: Amyotrophic lateral sclerosis
BPN: Brachial Plexus Neuritis
CSF: Cerebrospinal Fluid
EMG: Electromyography
GLP: 1-Glucagon-Like Peptide-1
HNA: Hereditary Neuralgic Amyotrophy
LP: Lumbar Puncture
MRI: Magnetic Resonance Imaging
MS: Multiple Sclerosis
NAION: Nonarteritic Anterior Ischemic Optic Neuropathy
NCS: Nerve Conduction Studies
PCP: Primary Care Physician
PCR: Polymerase Chain Reaction
PT: Physical Therapy
SNAP: Sensory Nerve Action Potential
STD: Sexually Transmitted Disease
